# E-Sem3DGS: Monocular Human and Scene Reconstruction via Event-Aided Semantic 3DGS

**DOI:** 10.3390/s26010188

**Published:** 2025-12-27

**Authors:** Xiaoting Yin, Hao Shi, Kailun Yang, Jiajun Zhai, Shangwei Guo, Kaiwei Wang

**Affiliations:** 1 State Key Laboratory of Extreme Photonics and Instrumentation, National Engineering Research Center of Optical Instrumentation, Zhejiang University, Hangzhou 310027, China; yinxiaoting@zju.edu.cn (X.Y.); jiajunzhai@zju.edu.cn (J.Z.); shangwei@zju.edu.cn (S.G.); 2 School of Electrical and Electronic Engineering, Nanyang Technological University, Singapore 639798, Singapore; 3 School of Artificial Intelligence and Robotics, National Engineering Research Center of Robot Visual Perception and Control Technology, Hunan University, Changsha 410082, China; kailun.yang@hnu.edu.cn

**Keywords:** 3D reconstruction, 3D Gaussian Splatting, event-based vision

## Abstract

Reconstructing animatable humans, together with their surrounding static environments, from monocular, motion-blurred videos is still challenging for current neural rendering methods. Existing monocular human reconstruction approaches achieve impressive quality and efficiency, but they are designed for clean intensity inputs and mainly focus on the foreground human, leading to degraded performance under motion blur and incomplete scene modeling. Event cameras provide high temporal resolution and robustness to motion blur, making them a natural complement to standard video sensors. We present E-Sem3DGS, a semantically augmented 3D Gaussian Splatting framework that leverages hybrid event-intensity streams to jointly reconstruct explicit 3D volumetric representations of human avatars and static scenes. E-Sem3DGS maintains a single set of 3D Gaussians in Euclidean space, each endowed with a learnable semantic attribute that softly separates dynamic human and static scene content. We initialize human Gaussians from Skinned Multi-Person Linear (SMPL) model priors with semantic values set to 1 and scene Gaussians by sampling a surrounding cube with semantic values set to 0, then jointly optimize geometry, appearance, and semantics. To mitigate motion blur, we derive optical flow from events and use it to supervise image-based optical flow between rendered frames, enforcing temporal coherence in high-motion regions and sharpening both humans and backgrounds. On the motion-blurred ZJU-MoCap-Blur dataset, E-Sem3DGS improves the average full-frame PSNR from 21.75 to 32.56 (+49.7%) over previous methods. On MMHPSD-Blur, our method improves PSNR from 25.23 to 28.63 (+13.48%).

## 1. Introduction

Reconstructing photorealistic, animatable human avatars, together with their surrounding static environments, from sensory input is a pivotal challenge in computer vision, with transformative applications in extended reality (XR), gaming, and visual try-on [1,2,3,4]. Recent advances in neural rendering integrate body articulation into Neural Radiance Fields (NeRFs) [5,6,7,8] and point-based rendering such as 3D Gaussian Splatting (3DGS) [9,10,11,12], enabling high-fidelity reconstruction of clothed human geometry and appearance from sparse or monocular videos. While these methods can utilize 3D volumetric representations to render high-quality human avatars under novel poses, they typically assume clean intensity inputs and mainly focus on the foreground human, often leaving static scenes under-modeled and degrading in the presence of motion blur [13].

Three-Dimensional Gaussian Splatting (3DGS) has emerged as an efficient alternative to NeRFs, offering fast inference and high-fidelity rendering with reduced computational cost [10]. Methods such as 3DGS-Avatar [9] and ASH [11] leverage 3DGS to render animatable human avatars by integrating articulated deformation with Multi-Layer Perceptrons (MLPs) or Convolutional Neural Networks (CNNs) for real-time performance. HUGS [12] extends this line of work to jointly model humans and static backgrounds by employing two separate sets of 3D Gaussians—one for the human and one for the scene—yet guided by precomputed human foreground masks [14,15]. However, maintaining dual Gaussian sets increases representation and optimization complexity, and the reliance on 2D foreground masks becomes problematic under motion blur, where mask predictions are noisy and misaligned. This limits scene realism and robustness in immersive applications such as augmented reality (AR) [1,2].

Motion blur is a common artifact in monocular videos of fast-moving subjects, introducing temporal ambiguities that challenge consistent reconstruction of both geometry and appearance across frames [16]. Intensity-based deblurring methods such as MPR [17] and NAFNet [18] attempt to restore sharp frames, but they struggle in highly dynamic scenes with complex motion patterns [19]. Event cameras, which asynchronously capture per pixel brightness changes, naturally complement standard video sensors by providing high-temporal-resolution data that is robust to motion blur [20]. Hybrid event-intensity approaches, including EFNet [19] and D2Net [21], improve deblurring by fusing event streams and intensity images, yet they are typically formulated as 2D pre-processing modules that are decoupled from downstream 3D reconstruction and have limited generalization across diverse scenes. In human pose estimation, EventHPE [13] demonstrates that event-derived optical flow can drive high-precision 3D human pose and shape estimations under motion blur, highlighting the potential of event-based motion cues for dynamic 3D reconstruction.

**Figure 1:**
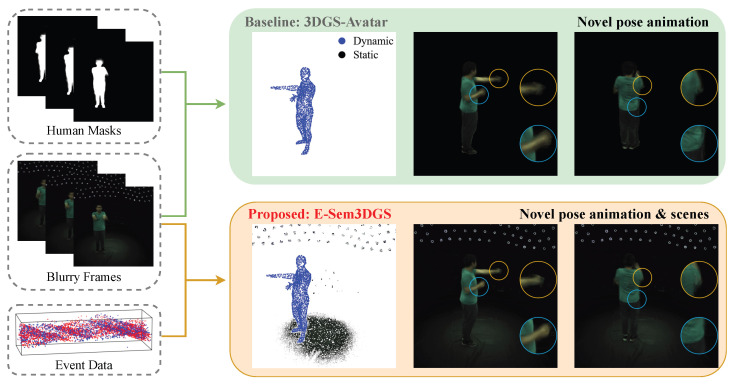
**Comparison between the baseline 3DGS-Avatar [9] and our E-Sem3DGS.** While the baseline relies on human masks and suffers from motion-blur artifacts in fast-moving scenes, our method integrates event data and semantic features, enabling improved human-body reconstruction and consistent static-scene modeling.

In this work, we address the joint reconstruction of animatable humans and static scenes from monocular, motion-blurred videos by introducing E-Sem3DGS, a semantically augmented 3D Gaussian Splatting framework that leverages event-based optical flow. Building upon 3DGS-Avatar [9], E-Sem3DGS maintains a single set of 3D Gaussians, each endowed with a learnable semantic attribute that softly separates dynamic human content from static scene content within a unified representation, as illustrated in Figure 1. We initialize human Gaussians from Skinned Multi-Person Linear (SMPL) model priors with their semantic values set to 1 and scene Gaussians by sampling a surrounding cube with their semantic values set to 0, then jointly optimize geometry, appearance, and semantics. Unlike HUGS [12], which relies on separate Gaussian sets and precomputed foreground masks, our unified semantic representation simplifies the pipeline and avoids dependence on external segmentation under blur.

To mitigate motion blur, we integrate event-based optical flow supervision. Specifically, we derive optical flow from event streams and use it to supervise image-based optical flow between rendered images at consecutive time steps, with the loss selectively applied to regions exhibiting high motion magnitude. This design enforces temporal coherence in high-motion regions and sharpens both human and background reconstructions, in contrast to approaches that apply 2D deblurring as a separate pre-processing stage [17,18,19,21]. Compared to 3DGS-Avatar [9] and HUGS [12] cascaded with intensity-based [17,18] or event-intensity deblurring [19,21] methods, our approach achieves superior performance on the ZJU-MoCap-Blur [6] and MMHPSD-Blur [13] datasets. On motion-blurred ZJU-MoCap-Blur, for full-frame rendering encompassing both humans and scenes, E-Sem3DGS improves the average PSNR from 21.75 to 32.56, corresponding to a 49.7% relative gain over 3DGS-Avatar [9]. On MMHPSD-Blur, our method improves PSNR from 25.23 to 28.63, corresponding to a 13.48% gain over HUGS [12].

Compared with prior 3DGS-based methods that either require precomputed masks for human–scene separation or treat deblurring as a stand-alone 2D pre-processing step, E-Sem3DGS integrates semantic disentanglement and event-based motion cues directly into a unified 3D representation and optimization pipeline.

In summary, our main contributions are threefold:

- We propose E-Sem3DGS, a semantically augmented 3D Gaussian Splatting framework that unifies human and scene reconstruction within a single Gaussian representation, enabling efficient human–scene disentanglement and high-quality rendering from monocular videos under motion blur.
- We introduce an event-based optical flow supervision strategy that exploits event-derived flows to guide image-based flows between rendered frames, enhancing temporal consistency and mitigating motion blur in high-motion regions.
- We construct motion-blurred ZJU-MoCap-Blur and MMHPSD-Blur benchmarks and conduct extensive experiments, showing that E-Sem3DGS significantly outperforms strong baselines and state-of-the-art methods on both human and full-frame reconstruction in blurry scenes.

## 2. Related Work

### 2.1. Neural Human Rendering

Early photorealistic rendering and animation utilized complex multi-camera setups [22] and manual rigging of human body meshes [23,24]. Subsequent statistical body-shape models [25,26,27,28,29] facilitated the representation of diverse body shapes yet lacked fine details such as clothing, hair, and accessories. Neural Radiance Fields (NeRFs) [5] have transformed 3D reconstruction by modeling geometry and appearance for view synthesis from multi-view images without extensive setup. Originally developed for static scenes [30,31], NeRF has been adapted for dynamic human rendering by incorporating body encodings [32,33,34] or by learning a canonical NeRF representation and transforming camera rays from the observation space to the canonical space to retrieve radiance and density values from the canonical NeRF [7,35,36]. However, most NeRF-based methods, reliant on large MLPs, suffer from slow training (hours to days) and rendering (seconds) [9,12]. Optimized schemes, such as learning functions at grid points [37,38], hash encoding [31], or the elimination of learnable components [39,40], have been developed. Three-Dimensional Gaussian Splatting (3DGS) [10] offers an efficient alternative to NeRF, modeling scenes as sets of 3D Gaussians splatted onto the image plane via alpha blending. The field of 3D Gaussian-based avatar reconstruction [9,12,41,42] has rapidly advanced. However, most existing methods primarily focus on reconstructing human avatars in isolation, often neglecting the concurrent reconstruction of static background scenes [9,41,42]. HUGS [12] represents a state-of-the-art approach by maintaining separate Gaussian sets for the human and scene. However, this hard separation relies heavily on precomputed 2D foreground masks [14]. In motion-blurred scenarios, mask prediction becomes unreliable, leading to severe error propagation where human parts are misclassified as background and fail to deform. In contrast, E-Sem3DGS employs a unified representation with soft, learnable semantic attributes. We use semantics as a differentiable gating mechanism to functionally control the deformation pathway. This enables a self-correcting convergence behavior: even if points are initially misclassified, flow-based supervision can update their semantic attributes, dynamically “activating” their deformation capabilities. This unified representation simplifies optimization complexity by maintaining a single Gaussian set while ensuring geometric robustness against mask failures.

### 2.2. Deblurring Neural Rendering

Several methods [43,44,45] have been developed to adapt NeRF and 3DGS for the generation of sharp outputs from blurry inputs. Deblur-NeRF [43] pioneered deblurring in NeRF for blurry inputs during training, using a compact MLP to model spatially dependent blur kernels. Subsequent advancements leverage physical priors from the blurring process [44] and jointly optimize Gaussian parameters with the camera trajectory to enhance rendering quality for dynamic human reconstruction [45].

With the development of event cameras, some works [16,46,47] have optimized NeRF and 3DGS solely using event streams. Recent works also integrate events and images for 3D reconstruction to mitigate blur from extreme camera shake [48,49,50]. DE-NeRF [51] reconstructs deformable neural radiance fields for fast-moving objects using event streams and sparse sharp RGB frames. EvaGaussians [49] integrates event streams to explicitly model motion blur and guide deblurring reconstruction, jointly optimizing 3DGS parameters and camera motion for high-fidelity novel view synthesis. EaDeblur-GS [50] utilizes an Adaptive Deviation Estimator (ADE) network and novel loss functions to achieve sharp 3D reconstructions. However, regarding the utilization of event streams, these methods [49,50,51] predominantly rely on event generation models, minimizing the discrepancy between captured events and those simulated based on brightness changes. While rigorous, this strategy requires precise calibration of sensor parameters (e.g., contrast thresholds), limiting generalization across different devices and lighting conditions. Notably, ExFMan [52] introduces a neural rendering framework that reconstructs high-quality dynamic humans from monocular blurry videos by leveraging event camera data and velocity-aware losses to mitigate motion blur. However, this approach explicitly estimates a 3D velocity field from the deformation network’s derivatives. It relies heavily on the accuracy of this internal velocity estimation, which is prone to failure under complex, non-linear articulated motions, and its event supervision remains sensitive to sensor calibration. In contrast, our E-Sem3DGS adopts event-based optical flow as a robust intermediate representation. By supervising the motion field directly with external flow cues, we abstract away sensor-specific signal variations and provide explicit geometric guidance.

### 2.3. Video Deblurring Methods

Motion blur in monocular videos presents significant challenges for 3D human reconstruction due to its ill-posed nature [19,21]. Traditional intensity-based (RGB or grayscale) deblurring methods [53,54,55] estimate 2D blur kernels or leverage supervised deep learning with paired blurry–sharp datasets [17,18,56] to recover sharp frames. Event-intensity hybrid methods exploit the high temporal resolution of event cameras [19,21] to complement standard intensity data for motion deblurring. For 3D human reconstruction, these methods can serve as a two-stage baseline, first deblurring images, then reconstructing the 3D human model. However, their limited generalization across varied scenes and lack of human-specific priors often lead to failures in handling complex motion [16,52]. In contrast, our framework integrates event-based optical flow supervision to align rendered image flows and event-derived flows, emphasizing high-motion regions to provide explicit geometric cues, improving reconstruction accuracy.

## 3. Preliminaries

### 3.1. 3DGS-Avatar

3DGS-Avatar [9] introduces an efficient method for reconstructing animatable human avatars from monocular videos using 3D Gaussian Splatting (3DGS). It initializes a collection of 3D Gaussians ($\left\{G_{0}\right\}$) in a canonical space derived from an SMPL mesh [26] and transforms them to the observation space via non-rigid and rigid deformations. The non-rigid deformation module is expressed as follows:

(1) $$\left\{G_{d}\right\}=\Phi_{\psi_{nr}}\left(\left\{G_{c}\right\},z_{p}\right),\;$$

where $\phi_{\psi_{nr}}$ is the deformation network that maps the canonical position ($x_{c}$) and a latent code ($z_{p}$ [57]), which encodes SMPL pose and shape parameters, to the Gaussian’s position, scale, and rotation offsets, as well as a feature vector:

(2) $$\left(\Delta x,\Delta s,\Delta q,z\right)=\phi_{\psi_{nr}}\left(x_{c},z_{p}\right),\;$$

resulting in deformed Gaussians with $x_{d}=x_{c}+\Delta x$, $s_{d}=s_{0}\cdot \exp\left(\Delta s\right)$, and $q_{d}=q_{c}\cdot \left[1,\Delta q_{1},\Delta q_{2},\Delta q_{3}\right]$. The rigid deformation uses Linear Blend Skinning (LBS) [26]:

(3) $$\left\{G_{o}\right\}=\Phi_{\psi_{r}}\left(\left\{G_{d}\right\},{\left\{B_{b}\right\}}_{b=1}^{B}\right),\;$$

where a skinning MLP ($\phi_{\psi_{r}}$) predicts weights at position $x_{d}$ and ${\left\{B_{b}\right\}}_{b=1}^{B}$ are bone transformations. A neural color model is applied to generate view-dependent appearance from canonicalized viewing directions, a per Gaussian color feature, a pose-dependent feature, and a per frame latent code, while as-isometric-as-possible constraints on Gaussian positions and covariances enhance generalization to unseen poses.

### 3.2. Event-Based Optical Flow

Integrating event information into neural rendering techniques [16,46,47] commonly involves directly utilizing raw event data, often by deriving reconstruction losses from event generation models. However, this direct approach can be sensitive to diverse event sensor characteristics and varying acquisition environments. Inspired by EventHPE [13] and its ability to robustly extract motion information, we adopt an alternative strategy, inferring explicit geometric clues in the form of event optical flow. Specifically, EventHPE uses an unsupervised encoder–decoder Convolutional Neural Network (CNN)—namely, FlowNet [13,58]—to estimate optical flow from event frames. Its loss function combines a photometric term (warped image pixel differences) and a smoothness term (penalizing flow discrepancies).

In our method, this event-derived optical flow plays a crucial role. We apply this supervision primarily to regions exhibiting high motion intensity, i.e., areas where the event optical flow magnitude is significant. This selective application allows us to focus our deblurring efforts precisely where motion blur is most severe and where event data provides the most reliable motion estimates. By integrating these precise geometric constraints, our method significantly enhances the reconstruction of dynamic humans from blurry monocular videos, effectively mitigating severe motion blur.

## 4. Method

### 4.1. Overview

**Figure 2:**
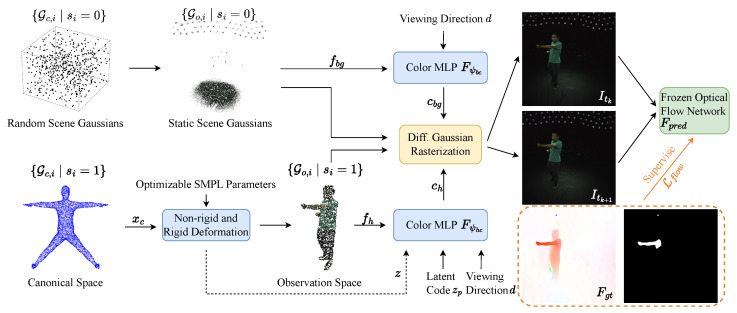
**Overview of our approach.** For a fixed hybrid sensor (e.g., Dynamic and Active-pixel Vision Sensor (DAVIS) camera [59]) capturing aligned event and intensity frame data, given a camera pose and human pose, E-Sem3DGS creates an avatar and renders human and scene images for specified human poses. The method represents human and scene regions with a set of Gaussians endowed with semantic attributes, where human Gaussians undergo deformation, while scene Gaussians remain static. Leveraging the event camera’s high temporal resolution, event-based optical flow supervision effectively mitigates dynamic blur from high-speed human motion during rendering.

This section introduces the E-Sem3DGS framework, which reconstructs dynamic human avatars and static scenes from monocular, jointly calibrated blurry videos and event data, along with provided SMPL parameters [26]. Our approach leverages event-aided semantic 3DGS to effectively mitigate motion blur and bypasses the need for human foreground masks by semantically distinguishing human and scene points. The method augments 3D Gaussians with a semantic logit vector initialized from the SMPL mesh for human Gaussians and random cubic sampling for scene Gaussians, with human Gaussians deformed via rigid and non-rigid transformations while scene Gaussians remain static. Deformed human Gaussians and static scene Gaussians are rendered through a single rasterization process supervised by a frozen flow network using event-based optical flow to address motion blur, as depicted in Figure 2. Semantic logits enhance human–scene segmentation (Section 4.2), a scene-color MLP ensures robust static-region appearance (Section 4.3), and event-based flow supervision mitigates dynamic blur (Section 4.4). Joint optimization of semantic Gaussians, human deformation, skinning, event-based flow, and color networks is conducted to reconstruct humans and scenes from blurry video inputs and event data (Section 4.5).

### 4.2. Semantic 3D Gaussians

We introduce semantic attributes to the 3D Gaussian primitives to enable explicit segmentation of dynamic human bodies and static scenes. Each 3D Gaussian ($G_{c,i}$) in the canonical space, defined by its mean $x_{i}$, scaling factor ($s_{i}$), rotation quaternion ($q_{i}$), opacity ($\alpha_{i}$), and color features ($f_{i}$), is augmented with a semantic logit vector ($l_{i}\in R^{K}$, where K=2 represents the background (0) and human (1) classes).

The semantic logits ($l_{i}$) are initialized based on input labels. Given a point cloud with *N* points and corresponding labels ($l\in {\left\{0,1\right\}}^{N}$), we initialize the logits as follows:

(4) $$l_{i,k}=\left\{\begin{array}{cc} \lambda_{\mathrm{logit}} & \mathrm{if}\;k=l_{i},\\ -\lambda_{\mathrm{logit}} & \mathrm{otherwise}, \end{array}\right.\;$$

where $\lambda_{\mathrm{logit}}=3.0$ is a scalar hyperparameter that controls the initial class confidence, ensuring high probability for the correct class. The semantic probabilities are computed via a softmax activation:

(5) $$P\left(k|l_{i}\right)=\mathrm{Softmax}{\left(l_{i}\right)}_{k}=\frac{\exp(l_{i,k})}{\sum_{j=1}^{K}\exp\left(l_{i,j}\right)},\;$$

where $l_{i}$ is a learnable parameter optimized during training. The predicted class for each Gaussian is obtained as

(6) $$s_{i}=\arg\max_{k}P\left(k|l_{i}\right),$$

and segmentation masks are derived as $m_{i,h}=\left(s_{i}=1\right)$ for the human class and $m_{i,\mathrm{bg}}=\left(s_{i}=0\right)$ for the background class, identifying points belonging to each class. Semantic attributes are integrated into the 3DGS pipeline, where human points are deformed from canonical to observation space using rigid and non-rigid transformations, then rasterized alongside background points for unified rendering of both parts.

### 4.3. Scene-Color MLP

We introduce a scene-color MLP to model static background appearance in monocular video to achieve higher expressiveness, flexibility, and robustness to noise or blur data compared to traditional spherical harmonics (SH) methods. For scene Gaussians ($\left\{G_{c,i}|s_{i}=0\right\}$), the MLP takes the feature vector ($f_{bg}\in R^{32}$) and the SH basis ($\gamma (\hat{d})$) of the viewing direction ($\hat{d}$) as input, predicting the appearance colors via

(7) $$c_{bg}=F_{\psi_{bc}}\left(f_{bg},\gamma \left(\hat{d}\right)\right),\;$$

where $F_{\psi_{bc}}$ is an MLP with one 64-dimension hidden layer and $\hat{d}$ is the canonicalized direction derived from the relative position of the 3D Gaussian and the camera center. This approach enables fine-grained, non-linear color modeling and leverages end-to-end optimization for improved reconstruction quality while ensuring robust color prediction for static regions.

### 4.4. Event-Flow Supervision

To enhance the reconstruction of motion-blurred human bodies in monocular videos, we incorporate event-based optical flow [13] as a supervisory signal, leveraging the high temporal resolution of event cameras. We employ a lightweight, frozen flow network—either SPyNet [60] or MaskFlowNetS [61]—to predict optical flow ($F_{\mathrm{pred}}\in R^{B\times H\times W\times 2}$) from pairs of rendered frames ($I_{t_{k}},I_{t_{k+1}}\in R^{B\times 3\times H\times W}$, where $t_{k}$ denotes the current time step in the frame sequence ($\left\{I_{t_{0}},\ldots ,I_{t_{k}},I_{t_{k+1}},\ldots ,I_{t_{n}}\right\}$)). The flow network, denoted as $F_{\psi_{f}}$, is defined as follows:

(8) $$F_{\mathrm{pred}}=F_{\psi_{f}}\left(I_{t_{k}}^{r},I_{t_{k+1}}^{r}\right),\;$$

where the frames are resized to a resolution scale of r=0.25 via bilinear interpolation to reduce computational cost, i.e.,

(9) $$I_{t_{k}}^{r}=R_{r}\left(I_{t_{k}}\right),\;I_{t_{k+1}}^{r}=R_{r}\left(I_{t_{k+1}}\right),\;$$

and $R_{r}$ is the bilinear resize operator.

The predicted flow ($F_{\mathrm{pred}}$) is supervised by event-based optical flow to enhance the rendering quality of fast-moving human body parts in motion-blurred monocular videos. The flow loss is defined as follows:

(10) $$L_{\mathrm{flow}}=\frac{1}{\left|M\right|}\sum_{p\in M}\left({\left|F_{\mathrm{pred}}\left(p\right)-F_{\mathrm{gt}}^{r}\left(p\right)\right|}_{1}+\epsilon \right),\;$$

where $M=\{p|\;∥F_{\mathrm{gt}}^{r}\left(p\right){∥}_{2}>\tau \}$ is a mask selecting pixels with significant flow magnitude, $\epsilon =10^{-8}$ ensures numerical stability, and ${\left|\;\cdot \;\right|}_{1}$ denotes the L1 norm of the vector difference. This masked L1 loss focuses supervision on regions with substantial motion, enhancing robustness to motion blur.

This supervision strategy is grounded in the premise that, despite originating from different modalities, both flows represent the same underlying physical velocity field. Consequently, minimizing their discrepancy enforces 2D motion consistency on the image plane. While this acts as geometric guidance for the projected deformation rather than strict 3D consistency under complex self-occlusions, it effectively provides structural constraints where intensity data is ambiguous. Moreover, the employment of flow networks acts as a robustness filter, abstracting away sensor noise and contrast threshold sensitivities to focus optimization on the correction of geometric deviations. Note that our framework is modular: while we employ specific pre-trained models in our experiments, the flow estimation module is compatible with general-purpose event optical flow networks (e.g., E-RAFT [62]), ensuring broad applicability across different datasets.

### 4.5. Optimization

We jointly optimize the 3D semantic Gaussians ($G_{c}$), comprising canonical human and scene Gaussians, along with human deformation, skinning, color networks for human modeling [9], and the scene-color MLP ($\psi_{bc}$) (Section 4.3), using event-based optical flow supervision with a frozen flow network to reconstruct dynamic human avatars and static scenes from blurry monocular videos and event data. The optimization is driven by a loss function combining (1) L1 loss for alignment of rendered and ground-truth images, (2) event-based flow loss (Section 4.4) for motion supervision, (3) perceptual loss [9] to provide robustness to local misalignments, (4) skinning loss based on SMPL priors, and (5) as-isometric-as-possible regularization losses for human Gaussians’ position and coherence. Note that the integration of event cues is achieved via gradient-based supervision rather than direct feature concatenation. The flow-loss gradients specifically guide the deformation in high-motion regions, effectively fusing temporal motion cues with the 3D geometry during backpropagation. Furthermore, structural integrity is maintained without external depth priors by employing skinning loss and as-isometric-as-possible regularization to penalize unphysical distortions.

## 5. Experiments

### 5.1. Datasets

**ZJU-MoCap-Blur.** Following 3DGS-Avatar [9], we select six sequences (377, 386, 387, 392, 393, and 394) from the ZJU-MoCap dataset [6] and generate motion-blurred images using Super-SloMo [63] to simulate realistic monocular video conditions. We select view “1” to focus on reconstructing both humans and scenes from a fixed viewpoint, aiming to minimize hardware costs and isolate high-speed human motion from camera ego-motion. Since this dataset lacks real event data, we employ a simulation strategy for event-based supervision. Specifically, the target optical flow is inferred from the sharp ground-truth images using the pretrained RAFT model [64]. This serves as a high-fidelity proxy for ideal event optical flow, which would naturally be free from motion-blur artifacts. To ensure training and test sets evenly sample the sequence and minimize train–test discrepancies arising from a single 360° human rotation, we apply an interleaved split. Specifically, we partition the sequence into consecutive blocks of 10 frames each, assigning the first 7 frames of every block to the training set and the subsequent 3 frames to the test set. This strategy ensures both comprehensive sampling across the entire sequence and a consistent train–test ratio for optical flow-based training. Human masks derived from RobustVideoMatting [15] enable comparisons with methods requiring human masks, with rendering quality assessed via PSNR, SSIM, and LPIPS metrics for both full images and human regions defined by bounding boxes.

**MMHPSD-Blur.** The MMHPSD dataset [13], captured using a single fixed CeleX-V event camera, provides event–grayscale image pairs, SMPL ground-truth parameters, and event-based optical flow. Specifically, the provided optical flow is inferred from event frames using the unsupervised FlowNet framework proposed in EventHPE [13,58]. Technically, this inference aggregates asynchronous events into multi-channel frames to explicitly encode polarity and high-temporal-resolution information into the motion estimation. Originally designed for 3D human pose estimation, the dataset is now extended for 3D reconstruction and rendering of dynamic human avatars in this work. To evaluate performance across diverse motion speeds and subjects, six sequences (s1g2t3, s5g1t1, s7g1t1, s10g3t4, s14g2t2, and s15g3t4) are selected. Motion-blurred images are generated using Super-SloMo [63] to replicate the visual effects of motion blur in monocular videos, with human masks derived via RobustVideoMatting [15] to enable comparisons with methods reliant on human segmentation.

### 5.2. Comparison with Baselines

Baseline methods include 3DGS-Avatar [9], which is a state-of-the-art method specifically designed for animatable human avatar rendering using 3D Gaussian Splatting, and HUGS [12], a prominent approach that simultaneously reconstructs and renders both animatable humans and static scenes within the 3DGS framework. For a fair comparison, the HUGS implementation adopts the official codebase [12], with scene point-cloud initialization modified from the original COLMAP-based approach [65,66] to random cubic sampling, denoted as HUGS^†^. To extend 3DGS-Avatar for simultaneous human and scene rendering, scalar semantic attributes are incorporated, with initialization combining the human body-mesh sampling and random cube sampling, setting initial semantic values to 0.5, referred to as 3DGS-Avatar*. Additionally, 3DGS-Avatar* and HUGS^†^ are cascaded with intensity-based deblurring methods (MPR [17] and NAFNet [18]) and Intensity+Event-based deblurring methods (EFNet [19] and D2Net [21]) for comparison. Deblurring results are input to 3DGS-Avatar* and HUGS^†^ for 3D reconstruction, denoted as Deblurring method + Reconstruction method.

**Figure 3:**
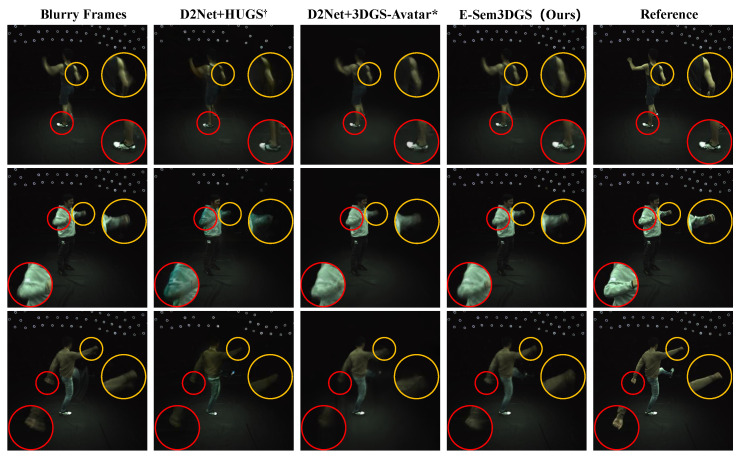
**Qualitative analysis on the ZJU-MoCap-Blur dataset.** Our E-Sem3DGS method, utilizing event information, significantly mitigates motion blur in dynamic regions (e.g., reduced arm-motion ghosting in the yellow-circled zoomed area and clearer shoe details in the red-circled area of the first row) compared to original blurred frames. Against tandem RGB-event deblurring approaches (D2Net + HUGS^†^ [12,21] and D2Net + 3DGS-Avatar* [9,21]), E-Sem3DGS demonstrates superior reconstruction fidelity in static background details, particularly in upper image regions.

First, we analyze the performance on the ZJU-MoCap-Blur dataset from multiple perspectives. **Quantitative Evaluation on Full-Frame Rendering:** Table 1 evaluates PSNR, SSIM, and LPIPS metrics across full images. 3DGS-Avatar, designed for human rendering, exhibits the lowest performance across sequences. Incorporating scalar semantic attributes in 3DGS-Avatar (3DGS-Avatar*) enables simultaneous human and scene rendering, boosting the PSNR from 21.75 to 25.47, with an improvement of 17%. Cascading with intensity deblurring enhances HUGS^†^ accuracy, whereas 3DGS-Avatar* remains unchanged, which is attributed to the limited discriminative power of scalar semantic attributes initialized at 0.5, constraining its responsiveness to deblurred inputs. Furthermore, D2Net with Intensity + Event methods further improves deblurring for HUGS^†^, while EFNet, trained on datasets with significant domain gaps relative to the current dataset, shows improvement over blurred inputs but underperforms compared to intensity-only deblurring. Our proposed method, integrating one-hot semantic attributes and event flow supervision, achieves the highest accuracy across all sequences. **Quantitative Evaluation within Human Bounding Boxes:** Table 2 reports average rendering metrics within human bounding boxes; the ZJU-MoCap-Blur dataset exhibits minimal background detail within these regions. 3DGS-Avatar*, with basic human–scene discrimination, increases the PSNR from 21.12 to 23.95 compared to 3DGS-Avatar, with the PSNR and SSIM trailing only the proposed method when cascaded with the Intensity + Event (D2Net) deblurring method. The proposed method yields the best PSNR, SSIM, and LPIPS values within human bounding boxes. **Qualitative Analysis:** In Figure 3, each column illustrates the input blurred image, D2Net + HUGS^†^, D2Net + 3DGS-Avatar*, the proposed method, and the reference image. Relative to the original blurry frames (with motion trailing highlighted in the first column’s yellow-circled zoomed inset), both the cascaded methods and the proposed method mitigate this artifact, demonstrating the benefit of event information in reducing dynamic blur. Compared to D2Net + HUGS^†^ and D2Net + 3DGS-Avatar*, the proposed method delivers sharper arm and hand contours, underscoring the advantage of event flow supervision over direct event deblurring cascades. Additionally, in the reconstruction of the static scene, HUGS^†^’s optimization proves challenging with random background initialization, and 3DGS-Avatar*’s scalar semantic attributes yield inadequate results (e.g., top regions), whereas the proposed method achieves a more realistic reconstruction.

**Figure 4:**
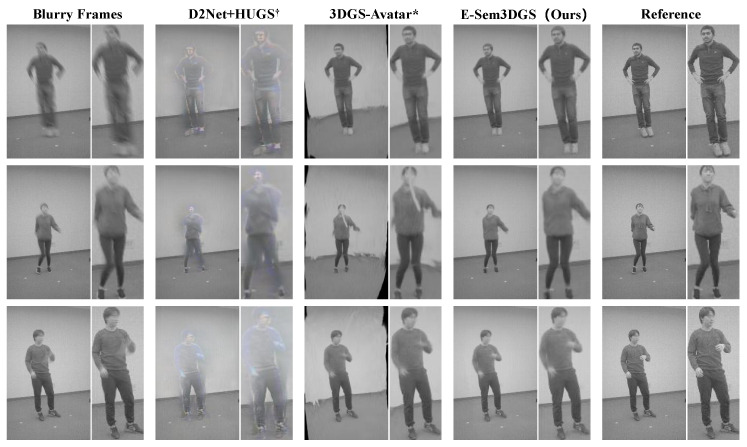
**Qualitative evaluation on the MMHPSD-Blur dataset.** For a fair comparison, HUGS [12] uses random background initialization instead of COLMAP [65,66], causing foreground and background points to interfere with human point optimization. 3DGS-Avatar* [9], with an initial semantic attribute of 0.5, misclassifies background points as human, degrading background rendering. Our E-Sem3DGS accurately reconstructs static scenes and leverages event information to enhance human body clarity compared to blurred images (see first row, columns one and four).

Then, we extend the evaluation to the performance on the MMHPSD-Blur dataset. Table 3 records per sequence results, with the proposed method attaining the highest PSNR and SSIM values, alongside the lowest LPIPS across most sequences. Unlike ZJU-MoCap-Blur, HUGS^†^ ranks second, with performance degrading when cascaded with deblurring methods due to the dataset’s complex background. Switching scene initialization from COLMAP to random sampling increases optimization difficulty for the two Gaussian groups (human + scene), where HUGS^†^’s reconstruction capability outweighs the impact of human blur severity. Table 4 presents quantitative results within human bounding boxes, with the proposed method achieving the best metric performance. Figure 4 displays the blurred image, HUGS^†^, 3DGS-Avatar*, the proposed method, and the reference image. As noted, HUGS^†^ (second column) maintains the human foreground and static scene background via two Gaussian groups, but random background initialization, unlike COLMAP’s facilitative structure, heightens optimization challenges, impairing human rendering quality. 3DGS-Avatar* outperforms HUGS^†^ for human rendering but misclassifies much of the background as human due to limited scalar semantic attribute capacity, causing co-deformation. The proposed method accurately reconstructs both humans and scenes, with event information supervision yielding clearer contours compared to the original blurred image.

### 5.3. Ablation Study

Ablation experiments are conducted on Subject 386 of the ZJU-MoCap-Blur dataset unless otherwise specified. As shown in Table 5, comparisons are made using only blurred image inputs, evaluating the effect of semantic attributes. 3DGS-Avatar [9], focused on human rendering, achieves a PSNR of 22.21 when tasked with simultaneous human and scene reconstruction due to its lack of scene modeling capability. Adding scalar semantic attributes enables differentiation of human and scene Gaussians, raising the PSNR to 33.47. Upgrading to one-hot semantic attributes further enhances this distinction, yielding the best rendering metrics, with a PSNR of 34.68.

Next, the integration of event information is ablated, as detailed in Table 6. Building on the blurred image baseline (last row of Table 5), the E2VID approach [67,68] converts events to grayscale images and blends them with blurry intensity images using a foreground human mask. This process disrupts image continuity and temporal consistency, lowering the PSNR from 34.68 to 29.34. Applying D2Net [21] for Intensity+Event deblurring modestly improves rendering, though the cascaded deblurring–reconstruction approach is limited by the deblurring model’s training scenarios. Incorporating event loss supervision [16,69], which computes loss from the log-intensity differences between pairs of rendered images compared to real events, effectively leverages event data to address motion blur, significantly enhancing rendering performance. While event loss supervision is effective, it often lacks generalizability across different event cameras due to varying sensor parameters (e.g., contrast thresholds) and inconsistent data formats (e.g., the polarity-free CeleX-V sensor in MMHPSD [13]). In contrast, optical flow [60,64,70,71] provides a more robust and universal representation of motion. Its effectiveness as an intermediate modality is well documented across a range of event-based downstream tasks, including Visual–Inertial Odometry (VIO) [72,73], keypoint detection [74], and human pose estimation [13]. By leveraging supervision from event-based optical flow to deblurring, our method achieves the highest evaluation metrics, improving the baseline with a gain of 6.2% in PSNR (36.82 vs. 34.68), a gain of 0.3% in SSIM (0.9837 vs. 0.9807), and a reduction of 42.8% in LPIPS (0.0345 vs. 0.0603).

Since the introduction of 3D Gaussian Splatting (3DGS) [10], the initialization problem has been a critical focus for researchers. In Table 7, we explore the impact of different initialization strategies on the reconstruction performance of our proposed method. When both human and scene points are randomly initialized, successful reconstruction is achieved. This is because our method leverages SMPL model parameters as a prior to optimize all learnable parameters, including 3D Gaussians, the deformation network, the skinning network, and the color network. Nonetheless, initializing only the human points based on the SMPL model while leaving scene points uninitialized leads to inferior reconstruction quality compared to random initialization of both human and scene points, as 3DGS relies on initial points for splitting and cloning. SMPL-based human initialization with random scene points improves performance over dual random initialization, with gray initialization of scene points to distinguish white human points, achieving the best rendering quality.

In our method, we employ semantic differentiation between human and scene Gaussians. Table 8 illustrates the impact of semantic initialization on reconstruction performance. Assigning an initial semantic value of 0.5 to all points (with human Gaussians corresponding to a semantic value of 1 and scene Gaussians corresponding to a semantic value of 0) enables successful reconstruction. However, this initialization significantly increases the learning complexity for the Gaussian model and associated networks. In contrast, initializing points on and inside the SMPL model surface with a semantic value of 1 and all other points with a semantic value of 0 results in superior rendering performance. Therefore, in our method, we initialize human Gaussians based on the SMPL model, randomly initialize scene Gaussians using a cubic volume, and assign a semantic value of 1 to Gaussians on and inside the SMPL model surface, with all other Gaussians assigned a value of 0. The performance gap suggests that while random initialization allows for reasonable convergence, the lack of explicit geometric guidance leads the optimization into suboptimal local minima with ambiguous semantic separation. In contrast, our SMPL-based initialization acts as a strong geometric prior, placing the system within a favorable basin of attraction. Furthermore, the stability during optimization is maintained by the synergy of loss constraints: the masked flow loss prevents dynamic parts from drifting into the static background, while the photometric intensity loss anchors the background, preventing it from drifting into the dynamic class. This mechanism effectively focuses the optimization on boundary refinement, though it remains sensitive to gross tracking errors where the prior is spatially decoupled from the image content.

**Figure 5:**
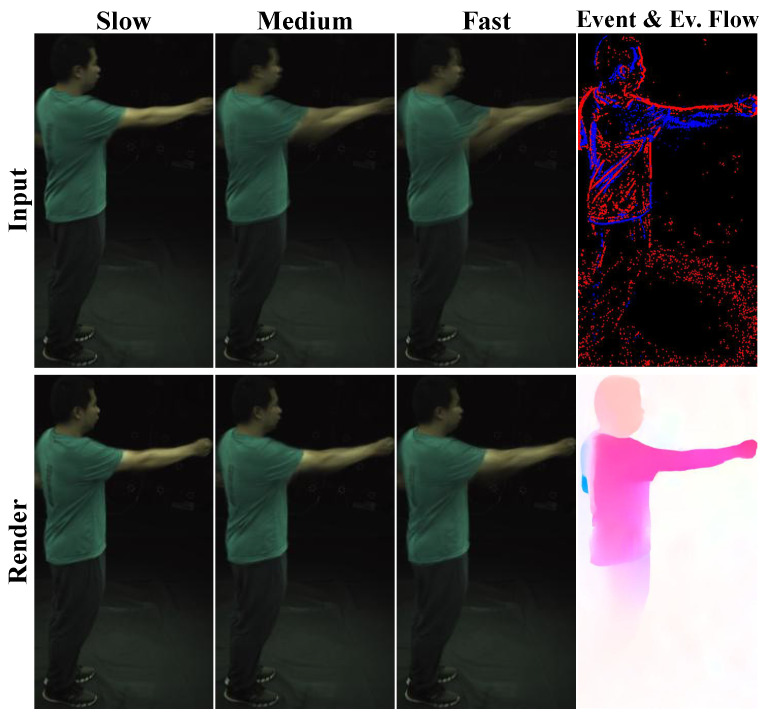
**Rendering performance under different blur levels.** This figure illustrates our method’s rendering results (bottom row) from blurry input images (top row) across *slow*, *medium*, and *fast* blur levels. Our method consistently generates sharper arm contours compared to the input, demonstrating effective blur mitigation across different motion speeds. By leveraging event-derived optical flow rather than raw event streams, our design provides more stable and sensor-agnostic motion cues, enabling robust deblurring across diverse acquisition conditions. The last column displays the raw event stream and the derived event-based optical flow, where color hue indicates motion direction and saturation indicates flow magnitude.

Figure 5 illustrates the rendering performance of our method under different blur levels (*slow*, *medium*, and *fast*), with the first row displaying input blurry images and the second row showing the corresponding rendering results. As the speed of the arm motion increases, the blur level in the arm region intensifies accordingly. Across varying blur levels, our method, by incorporating constraints from event optical flow, consistently produces arm contours that are sharper than those in the input blurry images. However, as the blur level increases, the arm edges in the rendering results become progressively more blurred, aligning with the changes observed in the input images.

To align with sustainable computing standards, we report our method’s efficiency. On a single NVIDIA RTX 3090 Ti GPU, training takes ∼35 min for a ∼400-frame sequence (256 × 256). Furthermore, inference operates at ∼19 FPS, demonstrating a favorable balance between high-fidelity reconstruction and computational cost.

## 6. Limitations and Future Work

Despite the effectiveness of our method in reconstructing both human bodies and scenes from a monocular hybrid sensor camera, several limitations remain. First, while optical flow supervision enhances reconstruction, it also introduces additional training complexity due to the computational overhead. However, during inference, rendering relies solely on the optimized 3D Gaussians and associated networks. Additionally, regarding hardware, the framework relies on hybrid sensors providing aligned intensity frames to ensure high-fidelity static scene reconstruction. Second, our evaluations are limited to indoor scenes due to the scarcity of real-world event camera datasets, leaving outdoor robustness under dynamic lighting and complex backgrounds unexplored. It is worth noting that despite these data constraints, our framework is theoretically extensible to broader settings. For varying camera trajectories, the optical flow supervision remains mathematically valid, as it naturally captures combined ego-motion and object motion. For multi-person scenes, our unified semantic representation can be extended by assigning distinct semantic identifiers to different subjects. Furthermore, the use of optical flow as an intermediate modality acts as a spatio-temporal filter, offering inherent robustness against sensor noise (e.g., hot pixels) and background clutter typical of unstructured environments. Furthermore, the limited pose space in training data may lead to suboptimal performance on unseen poses, especially for out-of-distribution motions.

To address these challenges, future work could explore the following directions. First, our method has demonstrated robust performance across diverse indoor and controlled lighting conditions. For a more comprehensive evaluation of its real-world applicability and robustness, future work could focus on testing its performance in more unconstrained settings, including complex outdoor environments characterized by dynamic natural light and intricate shadow variations, as well as scenarios involving moving cameras and multi-person interactions. Second, integrating generative models, such as diffusion-based approaches [75,76], could help augment the pose space and improve the model’s ability to generalize to previously unseen poses, improving performance in real-world scenarios. Finally, leveraging large-scale human motion datasets, such as AMASS [77], combined with zero-shot learning techniques, could enable the model to generalize across new identities, capturing a broader range of body shapes and appearances.

## 7. Conclusions

We presented E-Sem3DGS, a semantically augmented 3D Gaussian Splatting framework for joint reconstruction of animatable humans and static scenes from monocular intensity frames and collocated event streams. By maintaining a single set of 3D Gaussians endowed with learnable semantic attributes, our method explicitly disentangles dynamic human content from static backgrounds within a unified representation: human Gaussians are deformed through articulated networks, while scene Gaussians remain static. To cope with severe motion blur, we derive optical flow from events and use it to supervise image-based optical flow between rendered views, enforcing temporal coherence in high-motion regions and sharpening both geometry and appearance. Extensive experiments on synthetic and real-world motion-blurred datasets demonstrate that E-Sem3DGS consistently outperforms strong baselines and state-of-the-art methods on both human and full-frame reconstruction, achieving superior PSNR, SSIM, and LPIPS metrics. In future work, we plan to extend our framework to more complex interactions and multi-person scenarios and to further explore training on large-scale real event-camera datasets. 

## Figures and Tables

**Table 1:** Quantitative results on the ZJU-MoCap-Blur dataset.

Category	Method	Subject 377			Subject 386			Subject 387		
		PSNR	SSIM	LPIPS	PSNR	SSIM	LPIPS	PSNR	SSIM	LPIPS
Baselines	3DGS-Avatar [9]	21.53	0.1990	0.4060	22.21	0.2131	0.3888	21.78	0.2089	0.3972
	3DGS-Avatar* [9]	25.46	0.9494	0.1799	26.24	0.9524	0.1795	25.04	0.9405	0.1918
	HUGS^†^ [12]	26.03	0.9388	0.1094	28.53	0.9417	0.0938	24.93	0.9312	0.1107
Intensity-based Deblur	MPR [17] + 3DGS-Avatar* [9]	25.42	0.9485	0.1785	26.23	0.9512	0.1805	25.05	0.9406	0.1923
	MPR [17] + HUGS^†^ [12]	27.19	0.9455	0.0936	28.09	0.9428	0.0974	25.65	0.9354	0.0956
	NAFNet [18] + 3DGS-Avatar* [9]	25.42	0.9480	0.1787	26.26	0.9533	0.1784	25.09	0.9413	0.1891
	NAFNet [18] + HUGS^†^ [12]	27.26	0.9471	0.1012	28.92	0.9465	0.0913	25.13	0.9301	0.1187
Intensity + Event Deblur	EFNet [19] + 3DGS-Avatar* [9]	25.36	0.9446	0.1817	26.13	0.9465	0.1831	25.01	0.9368	0.1924
	EFNet [19] + HUGS^†^ [12]	26.47	0.9349	0.1227	27.92	0.9364	0.1022	25.17	0.9250	0.1107
	D2Net [21] + 3DGS-Avatar* [9]	25.48	0.9514	0.1794	26.29	0.9539	0.1798	25.13	0.9432	0.1903
	D2Net [21] + HUGS^†^ [12]	27.12	0.9483	0.1042	29.45	0.9494	0.0848	24.74	0.9289	0.1169
	Ours	**31.64**	**0.9779**	**0.0433**	**36.82**	**0.9837**	**0.0345**	**30.88**	**0.9721**	**0.0493**
**Category**	**Method**	**Subject 392**			**Subject 393**			**Subject 394**		
		**PSNR**	**SSIM**	**LPIPS**	**PSNR**	**SSIM**	**LPIPS**	**PSNR**	**SSIM**	**LPIPS**
Baselines	3DGS-Avatar [9]	21.68	0.2076	0.4129	21.52	0.1964	0.4097	21.77	0.2000	0.4012
	3DGS-Avatar* [9]	25.49	0.9433	0.2047	25.09	0.9385	0.2015	25.50	0.9407	0.1925
	HUGS^†^ [12]	26.03	0.9290	0.1381	27.34	0.9403	0.0987	27.31	0.9388	0.0943
Intensity-based Deblur	MPR [17] + 3DGS-Avatar* [9]	25.44	0.9421	0.2044	25.08	0.9382	0.2001	25.56	0.9420	0.1914
	MPR [17] + HUGS^†^ [12]	26.39	0.9326	0.1356	26.70	0.9359	0.1061	27.22	0.9372	0.0969
	NAFNet [18] + 3DGS-Avatar* [9]	25.43	0.9428	0.2045	25.09	0.9387	0.1995	25.51	0.9418	0.1910
	NAFNet [18] + HUGS^†^ [12]	25.92	0.9262	0.1356	26.20	0.9329	0.1146	24.67	0.9135	0.1522
Intensity + Event Deblur	EFNet [19] + 3DGS-Avatar* [9]	25.45	0.9399	0.2054	25.11	0.9363	0.2008	25.44	0.9380	0.1938
	EFNet [19] + HUGS^†^ [12]	26.56	0.9315	0.1283	25.78	0.9253	0.1319	24.91	0.9131	0.1395
	D2Net [21] + 3DGS-Avatar* [9]	25.52	0.9450	0.2037	25.11	0.9404	0.1997	25.53	0.9429	0.1922
	D2Net [21] + HUGS^†^ [12]	26.49	0.9367	0.1222	25.86	0.9293	0.1271	27.92	0.9419	0.0926
	Ours	**32.24**	**0.9752**	**0.0571**	**31.05**	**0.9712**	**0.0541**	**32.75**	**0.9746**	**0.0466**

* 3DGS-Avatar* denotes our enhanced version that incorporates scalar semantic attributes into 3DGS-Avatar (0 for background points and 1 for human body points). Initialization combines the human body mesh with random cube sampling, where semantic values are set to 0.5 initially. † Implementation note: Our HUGS implementation follows the official codebase [12] but modifies the scene point-cloud initialization from the original COLMAPbased approach to random sampling for consistency with comparative methods.

**Table 2:** Quantitative results within human bounding boxes on ZJU-MoCap-Blur.

Category	Method	Metrics		
		PSNR	SSIM	LPIPS
Baselines	3DGS-Avatar [9]	21.12	0.4984	0.3900
	3DGS-Avatar* [9]	23.95	0.8563	0.2685
	HUGS^†^ [12]	21.22	0.7521	0.2474
Intensity-based Deblur	MPR [17] + 3DGS-Avatar* [9]	23.96	0.8562	0.2648
	MPR [17] + HUGS^†^ [12]	21.35	0.7526	0.2456
	NAFNet [18] + 3DGS-Avatar* [9]	23.97	0.8578	0.2598
	NAFNet [18] + HUGS^†^ [12]	20.78	0.7346	0.2626
Intensity + Event Deblur	EFNet [19] + 3DGS-Avatar* [9]	23.87	0.8544	0.2742
	EFNet [19] + HUGS^†^ [12]	21.00	0.7381	0.2623
	D2Net [21] + 3DGS-Avatar* [9]	24.02	0.8594	0.2625
	D2Net [21] + HUGS^†^ [12]	21.30	0.7527	0.2420
	Ours	**24.66**	**0.8709**	**0.2123**

Note: The symbols * and ^†^ follow the same definitions as in Table 1.

**Table 3:** Quantitative results on the MMHPSD-Blur dataset.

Method	s1g2t3			s5g1t1		
	PSNR	SSIM	LPIPS	PSNR	SSIM	LPIPS
3DGS-Avatar [9]	7.12	0.3793	0.5006	7.27	0.3757	0.4822
3DGS-Avatar* [9]	17.25	0.7891	0.3905	16.16	0.7609	0.3665
HUGS^†^ [12]	27.49	0.8430	0.1155	24.08	0.8321	0.1170
MPR [17] + HUGS^†^ [12]	27.34	0.8414	0.1165	23.85	0.8233	0.1274
NAFNet [18] + HUGS^†^ [12]	26.92	0.8351	0.1151	23.68	0.8267	0.1183
D2Net [21] + HUGS^†^ [12]	27.36	0.8317	0.2099	23.70	0.8195	0.2100
Ours	**32.41**	**0.9378**	**0.1053**	**29.24**	**0.9250**	**0.1116**
**Method**	**s7g1t1**			**s10g3t4**		
	**PSNR**	**SSIM**	**LPIPS**	**PSNR**	**SSIM**	**LPIPS**
3DGS-Avatar [9]	6.69	0.3671	0.4978	6.54	0.3716	0.4929
3DGS-Avatar* [9]	14.50	0.7294	0.4379	13.19	0.6781	0.4580
HUGS^†^ [12]	26.20	0.8525	0.1129	22.89	0.8392	**0.1189**
MPR [17] + HUGS^†^ [12]	26.06	0.8452	0.1191	22.70	0.8376	0.1224
NAFNet [18] + HUGS^†^ [12]	25.61	0.8469	0.1162	22.81	0.8396	0.1216
D2Net [21] + HUGS^†^ [12]	25.96	0.8419	0.2058	22.04	0.8245	0.2218
Ours	**29.24**	**0.9390**	**0.1061**	**25.37**	**0.9153**	0.1398
**Method**	**s14g2t2**			**s15g3t4**		
	**PSNR**	**SSIM**	**LPIPS**	**PSNR**	**SSIM**	**LPIPS**
3DGS-Avatar [9]	6.89	0.3550	0.5025	6.62	0.3517	0.4975
3DGS-Avatar* [9]	17.18	0.7854	0.3970	12.64	0.6581	0.4733
HUGS^†^ [12]	27.17	0.8575	0.1031	23.52	0.8436	**0.1329**
MPR [17] + HUGS^†^ [12]	27.02	0.8537	0.1081	23.46	0.8416	0.1343
NAFNet [18] + HUGS^†^ [12]	27.03	0.8568	0.1036	23.50	0.8439	0.1348
D2Net [21] + HUGS^†^ [12]	26.93	0.8455	0.2100	23.50	0.8315	0.2341
Ours	**30.86**	**0.9458**	**0.0862**	**24.64**	**0.8998**	0.1502

Note: The symbols * and ^†^ follow the same definitions as in Table 1.

**Table 4:** Quantitative results within human bounding boxes on MMHPSD-Blur.

Method	Metrics		
	PSNR	SSIM	LPIPS
3DGS-Avatar [9]	6.73	0.2020	0.5736
HUGS^†^ [12]	17.76	0.5286	0.3722
MPR [17] + HUGS^†^ [12]	17.59	0.5196	0.3730
NAFNet [18] + HUGS^†^ [12]	17.45	0.5211	0.3683
D2Net [21] + HUGS^†^ [12]	17.34	0.5263	0.4445
3DGS-Avatar* [9]	21.52	0.7102	0.3938
Ours	**21.90**	**0.7389**	**0.3208**

Note: The symbols * and ^†^ follow the same definitions as in Table 1.

**Table 5:** Ablation study on semantic attributes in 3D Gaussians.

Method	Semantic		PSNR	SSIM	LPIPS
	Scalar	One-Hot			
3DGS-Avatar [9]	×	×	22.21	0.2131	0.3888
*w*/Scalar Semantic	✔	×	33.47	0.9791	0.0628
*w*/One-Hot Semantic	×	✔	**34.68**	**0.9807**	**0.0603**

Note: ✔ indicates the inclusion of the attribute, and × indicates its exclusion.

**Table 6:** Ablation study on different event utilization methods.

Case	PSNR	SSIM	LPIPS
Baseline	34.68	0.9807	0.0603
Baseline *w*/E2VID [67,68]	29.37	0.9626	0.0748
Baseline *w*/Intensity + Event Deblur [21]	34.73	0.9822	0.0643
Baseline *w*/Event Loss	36.63	0.9832	0.0361
Baseline *w*/Event Flow (Ours)	**36.82**	**0.9837**	**0.0345**

**Table 7:** Ablation study on 3DGS initialization strategies.

Body Init	Background Init	PSNR	SSIM	LPIPS
Random	Random	35.47	0.9808	0.0684
SMPL-based	N/A	26.23	0.9518	0.1770
SMPL-based	Random (White)	36.00	0.9821	0.0582
SMPL-based	Random (Gray)	**36.82**	**0.9837**	**0.0345**

Note: White = 1.0; gray = 0.5; N/A = no initialization.

**Table 8:** Comparison of 3DGS semantic initialization strategies.

Initialization Strategy	PSNR	SSIM	LPIPS
Random (all = 0.5)	29.35	0.9637	0.0924
SMPL-based binary masking *	**36.82**	**0.9837**	**0.0345**

* SMPL surface + interior = 1; others = 0.

## Data Availability

The original data presented in the study are openly available in Refs. [6,13].
